# Supplementary material for: Association between oral microbiome and seven types of cancers in East Asian population: a two-sample Mendelian randomization analysis
Source: Front Mol Biosci. 2023 Nov 21;10:1327893. doi: 10.3389/fmolb.2023.1327893 (PMC10702768; doi:10.3389/fmolb.2023.1327893)
Supplement: Supplementary file 1 [file DataSheet1.ZIP › Supplementary Materials/MR plots of saliva/saliva═╝/colorectal cancer/pheno.907_to_colorectal cancer_forest.pdf]

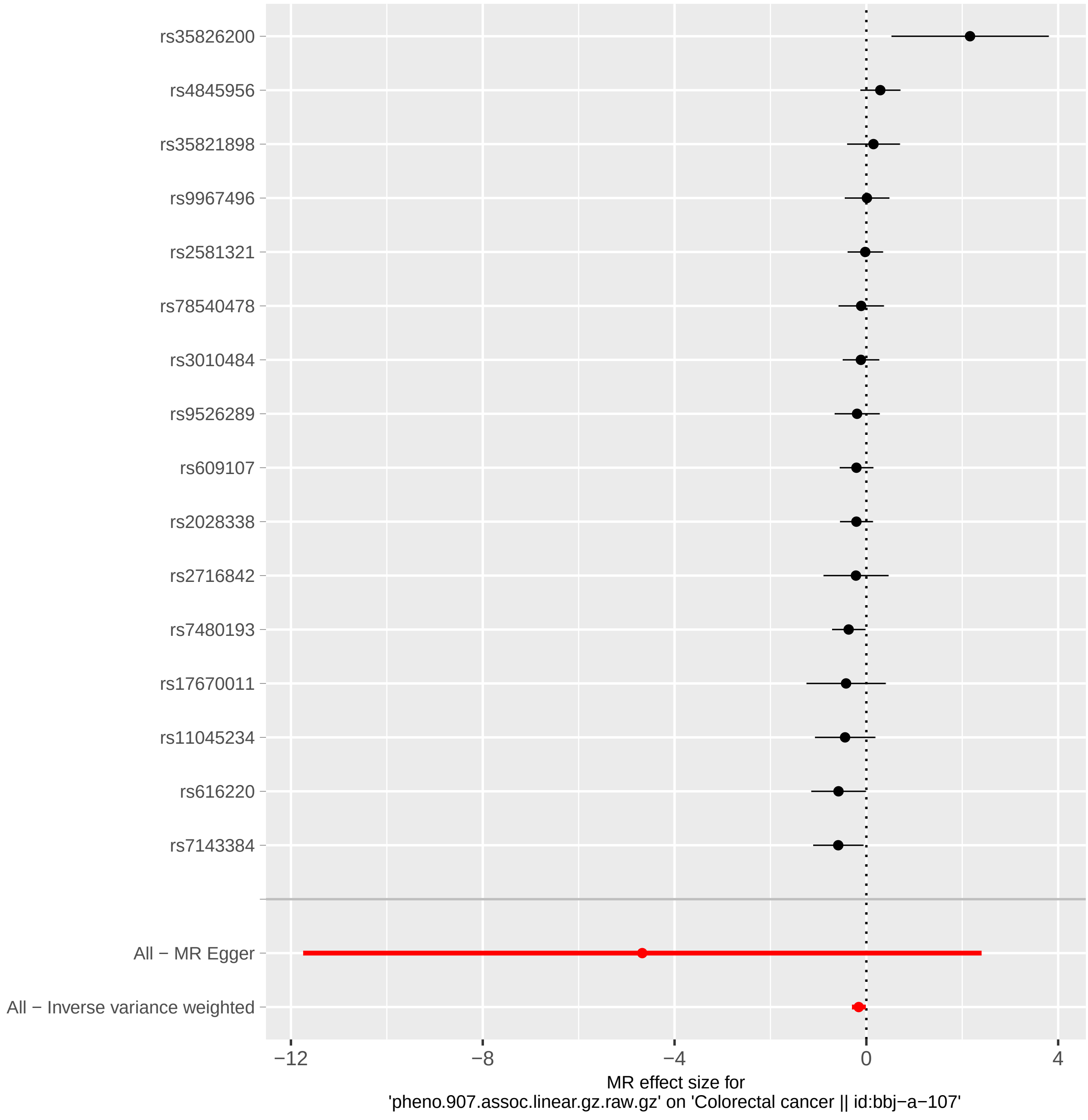

rs35826200
rs4845956
rs35821898
rs9967496
rs2581321
rs78540478
rs3010484
rs9526289
rs609107
rs2028338
rs2716842
rs7480193
rs17670011
rs11045234
rs616220
rs7143384
All − MR Egger
All − Inverse variance weighted
−12
−8
−4
0
4
MR effect size for
'pheno.907.assoc.linear.gz.raw.gz' on 'Colorectal cancer || id:bbj−a−107'
